# Orthogonal Cherenkov sound in spin-orbit coupled systems

**DOI:** 10.1038/srep11159

**Published:** 2015-06-17

**Authors:** Sergey Smirnov

**Affiliations:** 1 Institut für Theoretische Physik, Universität Regensburg, D-93040 Regensburg, Germany

## Abstract

Conventionally the Cherenkov sound is governed by *orbital* degrees of freedom and is excited by *supersonic* particles. Additionally, it usually has a *forward* nature with a conic geometry known as the Cherenkov cone whose axis is oriented *along* the *supersonic* particle motion. Here we predict Cherenkov sound of a unique nature entirely resulting from the electronic *spin* degree of freedom and demonstrate a fundamentally distinct Cherenkov effect originating from essentially *subsonic* electrons in two-dimensional gases with both Bychkov-Rashba and Dresselhaus spin-orbit interactions. Specifically, we show that the axis of the conventional *forward* Cherenkov cone gets a nontrivial *quarter-turn* and at the same time the sound distribution strongly localizes around this rotated axis being now *orthogonal* to the *subsonic* particle motion. Apart from its fundamentally appealing nature, the orthogonal Cherenkov sound could have applications in planar semiconductor technology combining spin and acoustic phenomena to develop, *e.g.*, acoustic amplifiers or sound sources with a flexible spin dependent orientation of the sound propagation.

Dating from the idea of a spin transistor^1^, systems with spin-orbit interactions have been attracting vasty interest because of a possibility to access the electronic spin degree of freedom by exclusively electric means and currently constitute a considerable part of contemporary spintronics^2^,^3^.

The interaction between the orbital and spin degrees of freedom is a relativistic effect and qualitatively it may be understood as a transformation of electric fields into magnetic fields in the rest system of an electron.

In two-dimensional semiconductor heterostructures two types of spin-orbit interaction are of particular importance. The first one is the Dresselhaus^4^ spin-orbit interaction due to the inversion asymmetry of the semiconductor crystal structure. The second one is the Bychkov-Rashba^5^ spin-orbit interaction appearing in asymmetric structures such as, *e.g.*, asymmetric quantum wells. In realistic systems both of these spin-orbit interactions are usually present and of comparable strengths which can be measured through, *e.g.*, Shubnikov-de Haas oscillations^6^,^7^, photocurrents^8^, optical monitoring of the spin precession^9^.

A qualitatively different class of condensed matter systems, where spin-orbit interactions are crucial, is the one of topological insulators^10^,^11^, the phase of matter where metallic edges coexist with an insulating bulk. The physics of the metallic edges is governed by helical states^12^,^13^. The surface helical states form Kramers pairs and the time reversal invariance leads to zero gap (or metallic) nature of these states while the states in the bulk are of finite gap (or insulating) character. The surface helical states are intimately linked to the bulk states and the time reversal invariance constrains the number of the Dirac points by even numbers.

Spin-orbit interaction mechanisms give rise to fascinating physical phenomena such as the intrinsic spin-Hall effect^14^,^15^ in semiconductors or the quantum spin-Hall effect^16^ in topological insulators, persistent spin helix^17^ as an interplay between the Bychkov-Rashba and Dresselhaus mechanisms in semiconductors or spin-transfer torques^18^ in topological insulators with applications to non-volatile memory.

The examples above spin around the electron dynamics. The spin-orbit physics has, however, another side related to the dynamics of the crystal lattice or phonons. Although the electron-phonon interaction has an orbital nature, the dynamics of the electronic orbital degrees of freedom is strongly affected by the electron spin degree of freedom in systems with spin-orbit interactions. Therefore, the impact of the electron spin on the phonon dynamics is of fundamental and practical interest. While the impact of phonons on the electron spin dynamics was extensively studied (*e.g.*, Dyakonov-Perel’^19^ mechanism or spin decay in quantum dots^20^,^21^), the role of the electron spin in the phonon dynamics was less explored.

One important aspect of the phonon dynamics is the acoustic Cherenkov effect, a counterpart of the optical Cherenkov effect^22^,^23^ where a medium emits a forward light cone under the action of superlight particles passing through this medium with velocities larger than the medium speed of light. Likewise, a medium emits a forward sound cone excited by supersonic particles whose velocity exceeds the medium sound velocity.

The presence of spin-orbit interaction mechanisms changes this picture. In two-dimensional semiconductor heterostructures with the Bychkov-Rashba spin-orbit interaction supersonic particles excite sound filling the forward Cherenkov cone and also the outward Cherenkov cone containing backward or anomalous (optical anomalous Cherenkov effect^24^, exists too but, in contrast to the present case, due to the spatial inhomogeneity) Cherenkov sound^25^. In other words, the Cherenkov cone angle which is, conventionally, between 0 and π/2 extends to π. On surfaces of three-dimensional topological insulators the Cherenkov sound is excited by helical particles which are always supersonic since the Dirac velocity is well above the sound velocity. In this case the geometry of the Cherenkov sound is also conic with the Cherenkov cone angle exceeding π/2, *i.e.*, the anomalous Cherenkov sound is generated in this case too. What makes the Cherenkov sound in topological insulators distinct is that at high energies it may strongly localize along certain directions^26^. Additionally, at low energies a magnetic field control of the Cherenkov sound in topological insulators may be of practical interest^27^.

The arc of vision above might suggests that spin-orbit interaction mechanisms enlarge the Cherenkov cone angle but, nevertheless, the very core of the Cherenkov physics remains unchanged: 1) the Cherenkov sound is still excited by supersonic particles; 2) its geometry still represents a single cone (although, the cone angle may exceed π/2); 3) the cone axis is still oriented along the direction of motion of the supersonic particle exciting the Cherenkov sound.

In the following we explore the acoustic Cherenkov effect in realistic two-dimensional semiconductor heterostructures with both Bychkov-Rashba and Dresselhaus spin-orbit interaction mechanisms of comparable strengths and demonstrate that peculiar coupling between the orbital and spin dynamics results in an acoustic Cherenkov effect of a unique nature fundamentally different from what has been known so far: 1) the Cherenkov sound is generated by essentially subsonic electrons; 2) the geometry of the Cherenkov sound excited by subsonic electrons represents a double cone; 3) the axis of the Cherenkov double cone gets a quarter-turn and, therefore, is orthogonal to the direction of motion of the subsonic particle exciting the Cherenkov sound; 4) the Cherenkov sound distributed within this rotated Cherenkov double cone is strongly localized around the cone axis or, in other words, the Cherenkov sound acquires an orthogonal nature.

A qualitative illustration of the orthogonal Cherenkov sound is shown in Fig. 1. In a two-dimensional electron gas with the Bychkov-Rashba and Dresselhaus spin-orbit interactions the electronic states are characterized (see below) by their chiralities λ and momenta 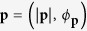, where 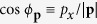, 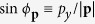. The energy spectrum consists of two branches, the upper (λ = +1) and the lower (λ = −1) ones. The isoenergy surfaces represent ellipses whose major axes is oriented along the line specified by the beams with the polar angle 3π/4 or 3π/4 + π. When the strengths of the two spin-orbit interaction mechanisms are comparable the major axes of these ellipses get much longer than the minor ones and the ellipses become extremely narrow. If an incident electron in the upper branch has a momentum **p**_*i*_ (here and below the subscripts *i* and *f* denote initial and final states, respectively) such that 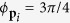, then, being subsonic at low energies, it will be unable to excite Cherenkov sound by virtue of intrachiral (λ_*i*_ = + 1 → λ_*f*_ = + 1) transitions. However, interchiral (λ_*i*_ = + 1 → λ_*f*_ = −1) transitions with 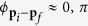 will not generate Cherenkov sound either because along these directions the electron energy changes very slowly and, despite the additional energy due to the energy gap between states with opposite chiralities, the electron energy loss is not compatible with its momentum change to emit a phonon. On the other side, interchiral transitions with 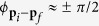 might generate Cherenkov sound (red areas in Fig. 1). Along these directions the electron energy changes faster and in combination with the energy gap between states with opposite chiralities such transitions may satisfy the energy and momentum conservation laws, **p**_*i*_ = **p**_*f*_  + **q**, *ε*_*i*_ = *ε*_*f*_  + *ħω*_**q**_ (**q** is the phonon momentum and *ħω*_**q**_ is its energy), see Fig. 1.

To quantitatively verify whether subsonic electrons may excite the Cherenkov sound within the above mentioned qualitative scenario as well as to explore its properties we briefly formulate below the computational scheme used to obtain the Cherenkov sound intensity. The single-particle Hamiltonian is:


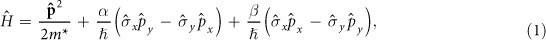


where m* is the electron effective mass, *α* and *β* are the strengths of the Bychkov-Rashba and Dresselhaus spin-orbit interactions, respectively. The eigenenergies of 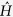 are


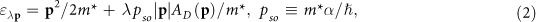



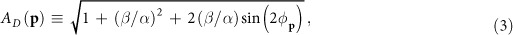


and its two component eigenspinors are 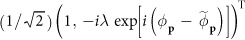, where 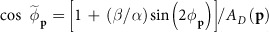, 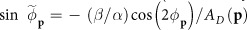.

The phonon part^28^ of the total Hamiltonian is


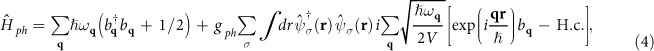


where for the acoustic phonons *ħω*_**q**_ = *c  *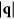 with *c* being the sound velocity. The first term in Eq. (4) describes the phonon gas in terms of the second quantized operators 
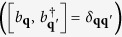
 while the second one describes the electron-phonon interaction of strength *g*_*ph*_ via the coupling to the electron field operators 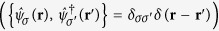.

Let us mention that the electron-phonon interaction in Eq. (4) is of the deformation potential type. In noncentrosymmetric crystals the piezoelectric electron-phonon coupling mechanism might also become important and in principle for a quantitative analysis both electron-phonon interactions should be considered. However, focusing on the qualitative aspect, we note that the orthogonal Cherenkov sound results from the general conservation laws of the energy and momentum and, therefore, its very existence can be predicted within a minimal model taking into account for simplicity only one electron-phonon interaction mechanism. It is our goal here to demonstrate within a minimal model the existence of an unusual Cherenkov effect which, in contrast to the known Cherenkov effects, is neither forward nor backward but, to some extent, is of an intermediate orthogonal nature. Moreover, taking into account possible applications in spintronic devices with a magnetic field control the deformation potential electron-phonon coupling mechanism may become dominant in InAs based structures^29^.

We follow the standard calculation^25^,^26^,^27^,^30^ to obtain the self-energy being the sum of the single-particle irreducible Feynman diagrams contributing to the electron Green’s function *G*_λ**p**_(*t* − *t*′) defined with respect to the physical vacuum 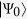, 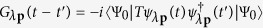, where the electronic field operators are in the Heisenberg representation. We calculate the self-energy in the second order in *g*_*ph*_ which is equivalent to the Fermi’s golden rule but the self-energy approach has a more systematic form easily generalized to derivations of the Cherenkov effect resulting from higher orders in *g*_*ph*_ as might be important in cases where the second order Cherenkov effect is absent. The dimensionless Cherenkov sound intensity as a function of the polar angle is obtained from the imaginary part of the self-energy,


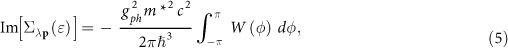


on the mass surface, *ε* = *ε*_λ**p**_, with λ = +1. It is given as the sum of the two terms, *W*(*ϕ*) = *W*_1_(*ϕ*) + *W*_2_(*ϕ*), originating, respectively, from intrachiral and interchiral transitions:


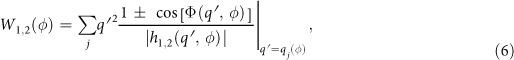


where *q*′ ≡ 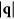 /m*c, *h*_1,2_(*q*′,*ϕ*) ≡ (8/m*c^2^)∂_*q*′_
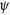
_1,2_(*q*′,*ϕ*), 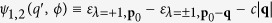, 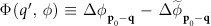, Δ*ϕ*_**p**_ ≡ *ϕ*_**p**_ − *ϕ*_0_, 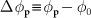, 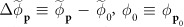, 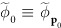, **p**_0_ is the initial electron momentum and the summation is over all the roots *q*_*j*_(*ϕ*) of the momentum-energy conservation equations,


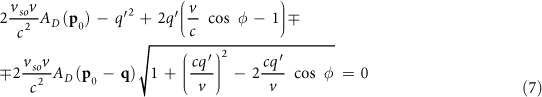


where *v*_*so*_ ≡ *p*_*so*_/m* is the anomalous spin-dependent velocity^31^ and *v* ≡ 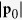/m*.

In Fig. 2 we show the total Cherenkov sound intensity, *W*(*ϕ*) = *W*_1_(*ϕ*) + *W*_2_(*ϕ*). For large *v*/*c* the Cherenkov sound is excited by supersonic electrons. In this case the sound is generated by both intrachiral and interchiral transitions and has the standard conic geometry where the cone axis has the same orientation *ϕ*_0_ as the electron momentum or group velocity and the cone angle extends up to π as expected^25^ in spin-orbit coupled systems for supersonic electrons. When *v*/*c* decreases the intrachiral contribution to the Cherenkov sound decays, the interchiral contribution starts to dominate and the sound intensity rapidly decreases along the standard cone axis, *i.e.*, in the vicinities of the angles *ϕ* = *ϕ*_0_ and *ϕ* = *ϕ*_0_ + π. For smaller *v*/*c* these vicinities grow into broad areas which eventually enclasp the whole space apart from the two enclaves around the two angles *ϕ* = *ϕ*_0_ ± π/2, *i.e.*, an orthogonal Cherenkov double cone forms as the most elegantly demonstrated by the blue curve for which *v*/*c* = 10^−5^ and 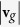/*c* ≈ 0.0814 (deep subsonic regime).

The actual distribution of the Cherenkov sound within the plane of the quantum well is best visualized using the polar coordinates as is done in Fig. 3. The parameters of the quantum well are the same as the ones used to obtain the data shown in Fig. 2 for *v*/*c* = 10^−5^ (deep subsonic regime, 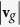/*c* ≈ 0.0814). In this representation the orthogonal nature of the Cherenkov sound is clearly revealed: the sound (dark areas) is localized mainly along the two directions orthogonal to the electron momentum or group velocity direction (red arrow) and consists of the left and right shoulders forming a narrow orthogonal Cherenkov double cone.

Finally, Fig. 4 demonstrates the total Cherenkov sound intensity for the case *α* = *β* for the same values of *v*/*c* as in Fig. 2. As one can see, here the Cherenkov sound has also an orthogonal nature. The localization of the Cherenkov sound around the direction perpendicular to **v**_*g*_ happens in this case faster because already for *v*/*c* = 10^−2^ one reaches the deep subsonic regime, 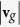/*c* ≈ 10^−2^. For even smaller values of *v*/*c* all the sound intensity curves collapse onto one curve representing the saturation limit corresponding to the linear dispersion of the initial electronic state *ε*_λ=+1,p_ for small momenta, 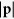 ≈ 0. Note also that for *α* = *β* the size of the localization domain also saturates so that the width of the peaks cannot be reduced by further decreasing *v*/*c*.

In conclusion we would like to mention that, in addition to its unique fundamental nature described above, the Cherenkov sound represents a general and realistic channel of the electron energy dissipation. Usually one assumes that at low energies the conventional Cherenkov dissipation is locked out since all particles are subsonic. Our analysis, however, shows that this is not the case in spin-orbit coupled systems and in practice one encounters the problem of energy losses also in the subsonic regime which might be important for phonon-limited low-field electron mobility^32^ being crucial for efficient high clock frequency and low power spintronics. Another practical aspect of the orthogonal Cherenkov sound is in diverse combinations of modern spintronics and acoustics to build such devices as acoustic amplifiers^33^,^34^,^35^ or sources of sound^36^ based on the acoustic Cherenkov effect with a flexible control of the sound direction determined by the electron spin dynamics in contrast to the more conventional manipulation mechanisms based on the orbital dynamics.

## Additional Information

**How to cite this article**: Smirnov, S. Orthogonal Cherenkov sound in spin-orbit coupled systems. *Sci. Rep.* **5**, 11159; doi: 10.1038/srep11159 (2015).

## Figures and Tables

**Figure 1:**
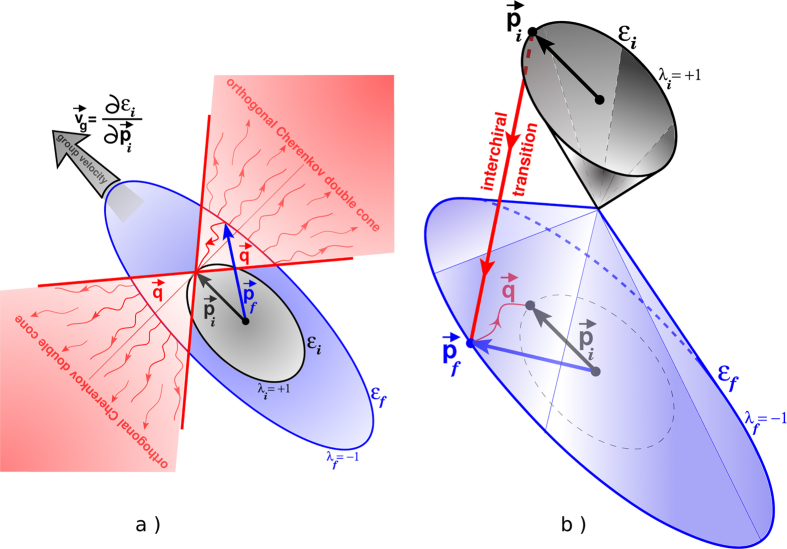
A qualitative illustration of the orthogonal Cherenkov sound in a two-dimensional electron gas with Bychkov-Rashba and Dresselhaus spin-orbit interactions. Part **a**) shows the process of the orthogonal Cherenkov sound generation in the plane of the two-dimensional electron gas. Part **b**) illustrates the corresponding interchiral transition using a three-dimensional representation of the electron spectrum (Eqs. (2) and (3)) at low energies and small momenta. A subsonic electron with the initial momentum **p**_*i*_ (black arrow) and chirality λ_*i*_ = + 1 (the corresponding energy is 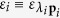) changes its state via the electron-phonon interaction. The electron final state is characterized by the momentum **p**_*f*_ (blue arrow) and chirality λ_*f*_ = −1 (the corresponding energy is 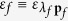). The electron group velocity in the initial state **v**_*g*_ = ∂*ε*_*i*_/∂**p**_*i*_ (wide black arrow) has the same direction as the initial momentum **p**_*i*_. The momentum and energy conservation laws admit the emission of a phonon (red wavy arrow connecting the vectors **p**_*i*_ and **p**_*f*_) within the double cone whose axis is orthogonal to **p**_*i*_ or **v**_*g*_. The phonon momentum and energy are **q** = **p**_*i* _−**p**_*f*_, *ħω*_**q**_ = *ε*_*i* _−*ε*_*f*_, respectively. Thus, instead of the normal forward or anomalous backward Cherenkov sound excited by supersonic particles within a cone whose axis is parallel to **p**_*i*_ or **v**_*g*_, the subsonic particles in spin-orbit coupled systems may excite a unique Cherenkov sound within a double cone whose axis gets a quarter-turn with respect to **p**_*i*_ or **v**_*g*_, as shown by multiple phonons (multiple red wavy arrows) within the red area indicating the Cherenkov double cone.

**Figure 2:**
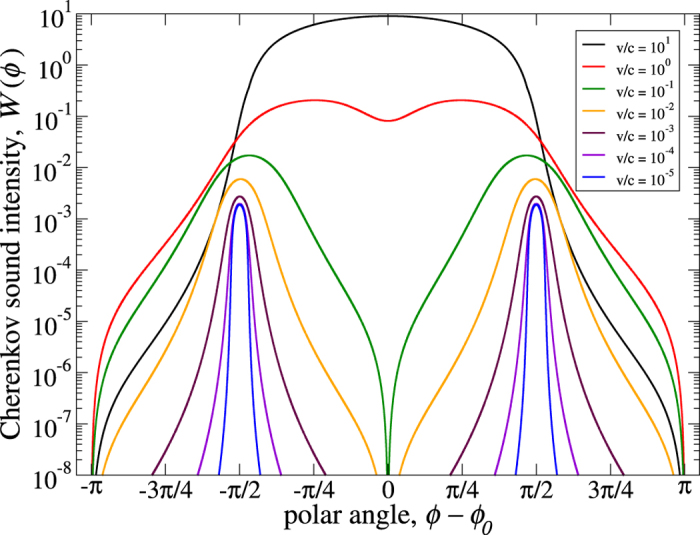
Cherenkov sound intensity as a function of the polar angle for different values of *v*/*c*. The parameters are for the two-dimensional electron gas formed in an InAs quantum well structure with *c* = 4.2 ⋅ 10^3^ m/s, m* = 0.038*m*_0_ (*m*_0_ is the free electron mass), *α* = 0.15 ⋅ 10^−11^ eV⋅m, *β*/*α* = 0.85. The sound is excited by electrons with λ = + 1 and momenta with orientation *ϕ*_0_ = 3π/4. The electron group velocity, **v**_*g*_ ≡ ∂*ε*_λ**p**_/∂**p**, has the same orientation.

**Figure 3:**
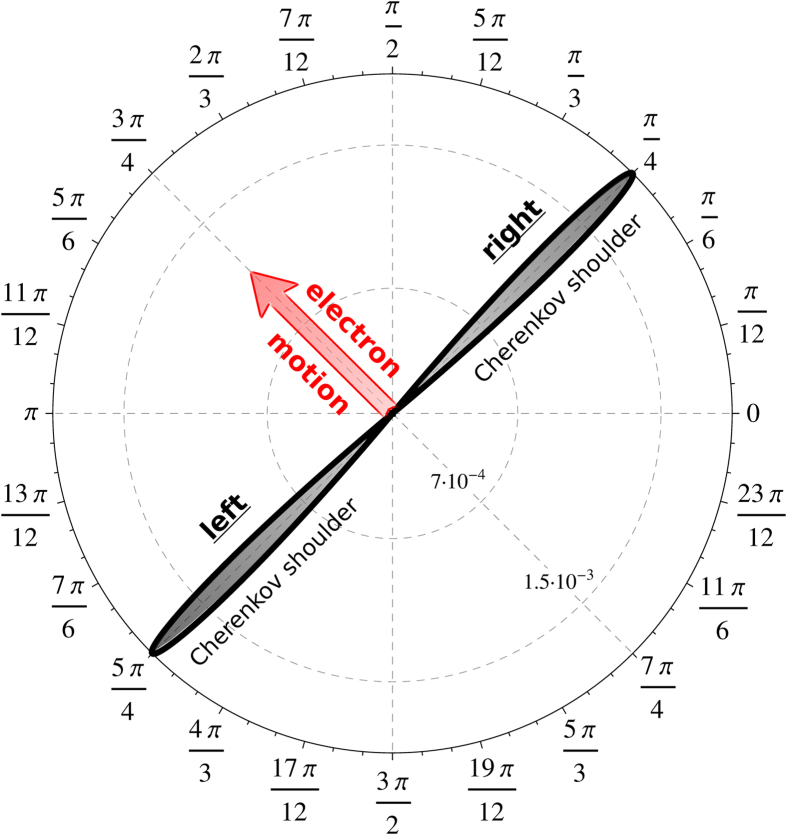
The two-dimensional distribution of the Cherenkov sound in the plane of an InAs quantum well. Here *v*/*c* = 10^−5^, *c* = 4.2 ⋅ 10^3^ m/s, m* = 0.038*m*_0_ (*m*_0_ is the free electron mass), *α* = 0.15 ⋅ 10^−11^ eV⋅m, *β*/*α* = 0.85. The sound is excited by electrons with λ = + 1 and momenta with orientation *ϕ*_0_ = 3π/4. The electron group velocity, **v**_*g*_ ≡ ∂*ε*_λ**p**_/∂**p**, has the same orientation.

**Figure 4:**
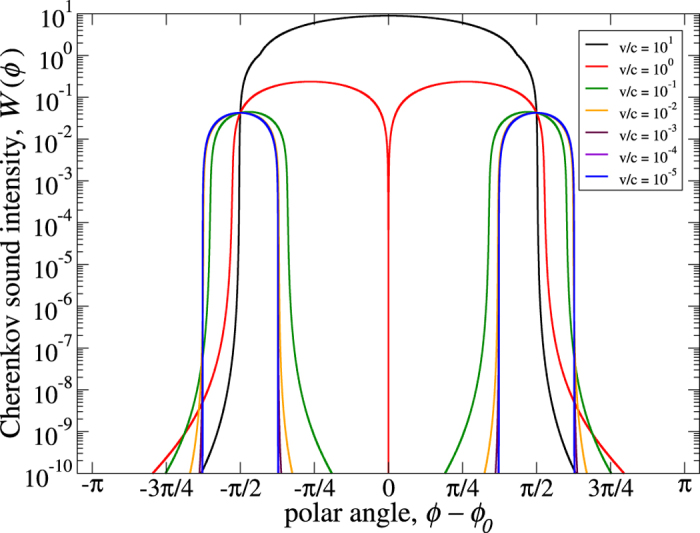
Cherenkov sound intensity as a function of the polar angle for different values of *v*/*c*. The parameters are for the two-dimensional electron gas formed in an InAs quantum well structure with *c* = 4.2 ⋅ 10^3^ m/s, m* = 0.038*m*_0_ (*m*_0_ is the free electron mass), *α* = 0.15 ⋅ 10^−11^ eV⋅m, *β* = *α*. The sound is excited by electrons with λ = + 1 and momenta with orientation *ϕ*_0_ = 3*π*/4. The electron group velocity, **v**_*g*_ ≡ ∂*ε*_λ**p**_/∂**p**, has the same orientation.
